# Lipoprotein(a) Induces Vesicular Cardiovascular Calcification Revealed With Single-Extracellular Vesicle Analysis

**DOI:** 10.3389/fcvm.2022.778919

**Published:** 2022-01-28

**Authors:** Maximillian A. Rogers, Samantha K. Atkins, Kang H. Zheng, Sasha A. Singh, Sarvesh Chelvanambi, Tan H. Pham, Shiori Kuraoka, Erik S. G. Stroes, Masanori Aikawa, Elena Aikawa

**Affiliations:** ^1^Center for Interdisciplinary Cardiovascular Sciences, Harvard Medical School, Brigham and Women's Hospital, Boston, MA, United States; ^2^Department of Vascular Medicine, Academic Medical Center, Amsterdam UMC, Amsterdam, Netherlands; ^3^Center for Excellence in Vascular Biology, Brigham and Women's Hospital and Harvard Medical School, Boston, MA, United States

**Keywords:** extracellular vesicles, cardiovascular calcification, lipoprotein(a), valve disease, atherosclerosis, inflammation

## Abstract

Lipoprotein(a) (Lp[a]) blood levels >50 mg/dL is a major cardiovascular disease risk factor in humans. Lp(a) associates with increased cardiovascular calcification, a critical pathology with no clinically available drug therapies. The mechanisms through which Lp(a) increases cardiovascular calcification risk remain undefined. We hypothesized that Lp(a) promotes the release of calcifying extracellular vesicles (EVs) that contribute to formation of microcalcification in cardiovascular tissues. Here, we show Lp(a) increased calcification in both primary human smooth muscle cells (SMCs) and valvular interstitial cells (VICs), potentially through inflammation-related mechanisms that were suppressed with E06 antibody that neutralizes pro-inflammatory oxidized phospholipids. Incubating human SMCs and VICs with Lp(a) altered the composition of EVs, increasing CD29^+^/tetraspanin^−^ microvesicle release, demonstrated with a tailored single-EV microarray assay that can distinguish multivesicular body-derived exosomes and plasma membrane budded microvesicles at a single-vesicle level. Lp(a) stimulation led to release of SMC and VIC EVs that readily calcified in acellular 3D-collagen hydrogels mimicking formation of ectopic microcalcification occurring in extracellular matrix of human atherosclerotic arteries and stenotic aortic valves. Our study mechanistically demonstrates that Lp(a) partially mediates cardiovascular calcification formation via inducing the release of calcifying EVs. Additionally, we provide a customized method to assess calcifying EVs at a single-vesicle level that can be more broadly applied to assist in quantitatively differentiating exosome and microvesicle EV subpopulations.

## Introduction

Ectopic calcification occurs in multiple diseases, as well as in advanced age. While all soft tissues in the body can calcify, a major unmet medical need is the paucity of anti-calcification therapies. Despite increasing cardiovascular disease mortality, cardiovascular calcification has no drug therapies available. Cardiovascular calcification occurs as vascular calcification in arteries and as valvular calcification in the heart. In arteries, microcalcification in atherosclerotic plaques may lead to plaque rupture (1), which in turn can lead to heart attack and stroke. In the heart, one of the leading causes of heart valve failure is macrocalcification, notably occurring in aortic valves causing aortic valve stenosis due to calcific aortic valve disease (CAVD). CAVD treatments are currently limited to invasive and costly surgical and transcatheter valve replacements (2, 3), which are inaccessible for millions of CAVD patients worldwide. Left untreated, progression of aortic valve stenosis leads to heart failure and death. A greater understanding of the molecular mechanisms driving cardiovascular calcification may led to development of first-in-class and life-saving therapies to treat these critical pathologies.

Multiple changes occur in cardiovascular cells that contribute to development of cardiovascular calcification (3). We and others have previously demonstrated a role of extracellular vesicles (EVs) in this process (4–10). Cardiovascular cells release calcifying EVs that get trapped in collagen extracellular matrix, aggregate, and form scaffolds that mineralize and grow forming vascular and valvular microcalcification (6, 10) that eventually coalesce into large macrocalcification. Triggers for this process are unclear but could involve inflammation and alterations in lipid metabolism that drive cardiovascular calcification (8, 11–13).

High blood levels of circulating lipoprotein(a) (Lp[a]), over 50 mg/dL, is a major risk factor for vascular and valvular disease (14). High Lp(a) levels are associated with increased coronary artery calcium scores (15), as well as increased calcification activity in CAVD patients (16). Lp(a) is a low-density lipoprotein (LDL)-like molecule containing apolipoprotein B, but unlike LDL also contains apolipoprotein(a). Statins, which reduce LDL do not substantially reduce Lp(a) or CAVD risk (2), supporting possible pathology-driving differences between Lp(a) and LDL. The physiologic functions of Lp(a) are unknown, and complicating Lp(a) mechanistic studies is that Lp(a) is not endogenously produced in commonly used laboratory animal models like rodents, with Lp(a) largely restricted to being produced in primates. Lp(a) mRNA targeting therapies are effective at lowering Lp(a) blood levels below the critical 50 mg/dL threshold (17) and are currently being assessed in clinical cardiovascular outcomes trials. How Lp(a) promotes cardiovascular calcification is unclear. Here we hypothesized that Lp(a) may contribute via the release of calcifying EVs. Therefore, we aimed to assess the mechanistic role of EVs in Lp(a)-mediated calcification, which was accomplished, in part, by utilizing a custom-tailored single-EV assay to quantitatively assess EV subpopulations. As we have shown similarities and differences between calcifying EVs released from valvular cells and vascular cells (10), we assessed the effects of Lp(a) on both cell types.

## Materials and Methods

### Tissue Culture

Human carotid artery tissues were obtained from endarterectomy procedures at Brigham and Women's Hospital (Institutional Review Board protocol #1999P001348). Human aortic valve tissues from donors undergoing valve replacement were obtained from Brigham and Women's Hospital (Institutional Review Board protocol #2011P001703). Written informed consent was obtained when applicable for the human tissues used in this study. All procedures performed involving human participants were in accordance with the 1964 Helsinki Declaration and its later amendments. Primary human coronary artery smooth muscle cells (SMCs) were obtained from Promocell (Heidelberg, Germany) and expanded in Promocell SMC Growth Medium 2 with Promocell SMC media supplement containing epidermal growth factor (0.5 ng/mL), insulin (5 μg/mL), basic fibroblast growth factor-β (2 ng/mL), and 5% fetal bovine serum. Primary human valvular intersitial cells (VICs) were obtained from human aortic valve tissue with collagenase digestion. Tissue-derived SMCs were confirmed as alpha smooth muscle actin-positive by FACS (18). Tissue-derived VICs were confirmed as vimentin positive by immunohistochemistry (19). Cells were cultured at 37 degrees Celsius (5% CO2, 90% humidity) and used between passages 1 and 5. Researchers were blinded to all patient clinical characteristics for the tissues and cell cultures used in this study. Human Lp(a) was commercially obtained (Lee Biosolutions Inc., Maryland Heights, MO; ≥ 95% purity by Helena SPIFE lipoprotein electrophoresis) and incubated with cells at a concentration of 10 ug/mL, a concentration previously demonstrated to induce VIC osteogenic differentiation (16). High Lp(a) (>50 mg/dL) and low Lp(a) (<50 mg/dL) serum from CAVD donors was previously described (16). Serum Lp(a) concentrations were measured by a commercial Lp(a) ELISA according to manufacturer's instructions (abcam #ab212165).

### Immunofluorescence

Human artery and valve tissues were cut into cryosections with 7 μm thickness and fixed in acetone. For microcalcification detection, cryosections were incubated with OsteoSense680 (1:100, Perkin Elmer, Waltham, MA), near-infrared based bisphosphonate calcium tracer, for 1 hour at room temperature; or for collagen detection, with CNA35-OG488 (1:50; provided by Carlijn V. C. Bouten, Eindhoven University of Technology), a collagen probe, for 1 h at room temperature prior to fixation. For Lp(a) uptake immunofluorescence, VICs were incubated with 10 ug/mL Lp(a) for 6 h prior to fixation, a time point that was experimentally determined using Western blot analysis to have maximal Lp(a) uptake. For cell immunofluorescence, cells were fixed in 4% paraformaldehyde. Two cryosections were analyzed per donor. Cryosections and cells were blocked in 4% serum, and permeabilized with 0.5% triton X-100 used for cell culture immunofluorescence (Thermo Fisher Scientific, Waltham, MA) and then incubated with CD63 (1:50, BioLegend #353021, Dedham, MA), CD29 (1:50, Biolegend #303016), apoliprotein(a) (1:50, Sigma MABS1284, Burlington, MA) or IgG negative control antibodies (abcam #ab172730, Cambridge, MA, Santa Cruz Biotechnology #sc2025, Dallas, TX). DAPI (Thermo Fisher Scientific) was used to stain nuclei. Immunofluorescence slides and cell culture wells were imaged using a confocal microscope A1 (Nikon Instruments Inc., Melville, NY). All images were processed with Elements 3.20 software (Nikon Instruments Inc.).

### Transmission and Density-Dependent Electron Microscopy

Transmission electron microscopy was performed at the Massachusetts General Hospital Program in Membrane Biology Electron Microscopy Core with a JEOL 1011 electron microscope. For EV analysis, 8 mL of conditioned media, was spun at 1,000 times gravity to remove cell debris and then the supernatant was centrifuged at 100,000 for 1 h at 4°C in a Beckman Coulter (Brae, CA) Optima MAX-XP Ultracentrifuge with an MLA-55 rotor to pellet EVs. EV pellets were fixed with 2% glutaraldehyde in 0.1 mol/L sodium cacodylate buffer. The fixative was removed, and EVs were rinsed with 0.1 mol/L sodium cacodylate buffer (Sigma) prior to imaging. For Lp(a) uptake cells were incubated with 10 ug/mL Lp(a) for 6 h prior to fixation, a time point that was experimentally determined using Western blot analysis to have maximal Lp(a) uptake. For immunogold electron microscopy, cells and Lp(a) standard were fixed in 4.0% paraformaldehyde with 0.2% glutaraldehyde (Sigma) in 0.1 mol/L sodium cacodylate buffer for 2 h at room temperature, and then overnight at 4°C before removing fixative and replacing it with 0.1 mol/L sodium cacodylate buffer. Samples were then incubated with a validated apolipoprotein(a) antibody (1:100, Sigma #MABS1284).

Density-dependent scanning electron microscopy was performed at University College London as previously described (10), with human carotid artery and aortic valve cryosections on glass slides that were secured to aluminum sample holders with carbon tape, and silver paint was applied to the area immediately surrounding each sample. Samples were then coated with 5 nm carbon (Quorum Technologies Turbo-Pumped Thermal Evaporators model K975X, Lewes, United Kingdom). Following coating, the samples were imaged on a scanning electron microscope (SEM Zeiss VP), operated at 10 kV, and equipped with both an inlens detector that recorded secondary electrons and a backscatter electron detector. Images were obtained by imaging a region in inlens mode and then subsequently imaging the same region in backscatter mode. Adobe Photoshop CC 2018 (San Jose, CA) software was used on stacked images, and the inlens image was assigned to the green channel whereas the backscatter image was assigned to the red channel.

### Proteomics

CAVD serum and Lp(a) standard samples were proteolyzed using the iST in-solution digestion kit (PreOmics GmbH, Germany) automated on the PreON robot (PreOmics): 1 μL serum sample was diluted in 9 uL phosphate buffer saline and then added to 40 uL LYSE buffer (PreOmics). 10 μg of Lp(a) protein standard was added to 40 μL LYSE buffer. The samples were trypsinized for 1 h following the manufacturer's instructions. Eluted peptides were dried in a speed vacuum (Eppendorf Vacufuge) and resuspended in 40 μL sample buffer (0.1% formic acid, in mass spectrometry grade water). Lp(a) standard peptide stock was diluted 50-fold and 2 μL analyzed, whereas 2 μL of the serum peptide stock were analyzed by mass spectrometry.

Data-dependent acquisition (DDA) mass spectrometry was acquired on the Orbitrap Fusion Lumos coupled to a heated EASY-Spray nanosource and a nanoLC1000 (Thermo Fisher Scientific). The peptides were subjected to first an Acclaim PepMap RSLC C18 trap column (75 μm X 20 mm), and then separated with a heated EASY-Spray column (45 degrees Celsius; 75 μm X 250 mm) (Thermo Fisher Scientific). Lumos was set to 120 K resolution, and the top N precursor ions in a 3 second cycle time (within a scan range of 400–1,500 m/z) were subjected to collision induced dissociation (CID, collision energy 30%; ion trap as the detector) for peptide sequencing. The parallelization feature was enabled (automatic gain control target, 1.0e5; maximum injection time, 35 ms). For serum peptides the gradient flow rate was 300 nL/min from 5 to 21% solvent B (acetonitrile/0.1% formic acid) for 75 min, 21 to 30% Solvent B for 15 min, followed by an additional 10 min of a solvent A5–B95% jigsaw wash. For the Lp(a) standard peptides the Lumos was set to 120 K resolution, and the top N precursor ions in a 3 second cycle time (within a scan range of 400–1,500 m/z) were subjected to higher energy collision energy dissociation (HCD, collision energy 30%; Orbitrap at resolutio*n* = 30 K as the detector) for peptide sequencing. The parallelization feature was enabled (automatic gain control target, 1.0e5; maximum injection time, 54 ms).

Mass spectral analysis. The DDA spectra were queried against the Human UniProt database (downloaded September 09, 2020; 96,816 entries) using the HT-SEQUEST search algorithm, via the Proteome Discoverer (PD) Package (version 2.2, Thermo Fisher Scientific), using a 10 ppm tolerance window in the MS1 search space, and a 0.02 Da fragment tolerance window for HCD and 0.6 Da for CID data (with trypsin as the enzyme). Methionine oxidation was set as a variable modification, and cysteine carbamidomethylation was set as a static modification. The peptide false discovery rate (FDR) of 1% was calculated using Percolator provided by PD. For quantification of proteins across patient samples the Feature Mapper node was used. For chromatographic alignment, the maximum retention shift was set to 10 min and mass tolerance 10 PPM. For feature linking and mapping, the retention time tolerance was set to 0 min and the mass tolerance 10 PPM, and the signal-to-noise threshold set to 5.

Lp(a) protein network (using Lp(a) standard proteins supported with at least three unique peptides) was generated with String: function protein association networks (string-db.org). Serum proteomics volcano plot was made using serum proteins identified with two or more unique peptides.

### SMCs and VICs *in vitro* Calcification

SMCs and VICs calcification induction was carried out by incubating 100% confluent SMCs and VICs in control M199 basal media (Thermo Fisher Scientific) with 10% fetal bovine serum and 1% penicillin/streptomycin, or M199 with the following calcification-inducing additions with or without 10 ug/mL Lp(a): 10 mmol/L β-glycerol phosphate (BGP; Sigma), 10 nmol/L vitamin D3 (Sigma), 10 nmol/L dexamethasone (Sigma), 0.8 mmol/L CaCl2 (Sigma). To induce calcification, 10 ug/mL Lp(a) was incubated with cells, as that concentration was previously demonstrated to induce VICs osteogenic differentiation (16). Cells were cultured for 2 weeks, changing media twice per week. Two weeks media incubation was used for calcification staining, as this time point showed clear calcification staining with Alizarin red under Lp(a) incubation conditions compared to an earlier one-week time point. For EV isolation, after 48 h in BGP media with and without Lp(a), cells were switched to calcifying media with exosome and lipoprotein-depleted serum for 24 h prior to collection to avoid serum EV and lipoprotein contamination. Conditioned media containing EVs was centrifuged at 1,000 times gravity to pellet any cell debris prior to further use. Calcification was assessed by 2% Alizarin red staining and quantified by extracting the stain with 100 mmol/L cetylpyridinium chloride (Thermo Fisher Scientific) for 1 h, and the sample absorbances were measured at 540 nm on a Molecular Devices SpectraMax M5 plate reader (San Jose, CA). For E06 oxidized phospholipid-neutralizing antibody experiments, 1 μg/mL E06 antibody (Avanti Polar Lipids, #330001S) was added with each media change.

### Real Time PCR

After 14 days in culture, RNA was isolated from SMCs and VICs using collected using TRIzol® reagent (Thermo Fisher Scientific) according to the manufacturer's instructions. cDNA was synthesized using 1 μg of total RNA with the qScript cDNA Synthesis Kit (Quantabio, Beverly, MA) according to manufacturer's protocol. qPCR reactions were performed using PerfeCTa® qPCR FastMix® II, ROX™ (Quantabio) and following Thermo Fisher Scientific Taqman primers: *ALPL* (#Hs01029144_m1, Fisher Scientific), *RPLPO* (#Hs99999902_m1, Fisher Scientific, used as housekeeping gene for *ALPL* mRNA levels normalization).

### Western Blot Analysis

Western blots for cell and EV lysates were performed by lysing cells and EV pellets in RIPA buffer with Halt protease inhibitor (Thermo Fisher Scientific), loading 10 μg total protein lysate, and using the following antibodies: ANXA1 (1:1,000, abcam #ab214486), ANXA2 (1:1,000, abcam #ab178677), ANXA6 (1:1,000, Santa Cruz Biotechnology #271859), β-actin (1:1,000, Novus #NB600-501, St. Louis, MO), calnexin (1:1,000, Cell Signaling Technologies #2679S, Danvers, MA), CD29 (1:1,000, BioLegend #303002), CD63 (1:1,000, abcam #ab8219), GAPDH (1:1,000, Santa Cruz Biotechnology #0411), GM130 (1:1,000, abcam #ab52649), histone H3 (1:1,000, abcam #ab176842), TOMM20 (1:1,000, abcam #ab205486). Blots were incubated with antibodies overnight at 4 degrees Celsius and imaged using with an Amersham ImageQuant 800 (Amersham, UK). Blots were stripped and reprobed for ANXA1, ANXA2, and GAPDH antibodies using Restore^TM^ Western Blot Stripping Buffer (Thermo Fisher Scientific). CD29 was used as a loading control for quantification of EV blots as the total CD29^+^ EV population was unchanged assessed by single EV microarray. GAPDH was used as a loading control for quantification of SMCs and VICs whole cell lysates.

### Nanoparticle Tracking Analysis

EVs diameters and abundances were determined by NanoSight LM10 (Malvern Instruments, Malvern, United Kingdom) nanoparticle tracking analysis. Conditioned cell culture media was collected after cells were incubated in M199 and BGP media with and without Lp(a). Conditioned media was centrifuged at 1,000 times gravity for 5 min to remove any cell debris. Supernatant was then diluted 1:10 in phosphate buffered saline, and the samples were injected continuously via a syringe pump, with five runs used to calculate means and standard deviations for each sample. The NanoSight camera gain was set at a constant value of 9, and threshold values for EV detection was set to 2. The EVs size and concentration from the collected data was averaged to obtain the distribution in each sample.

### Single-EV Microarray

SMCs and VICs were cultured for 48 h in M199 and BGP containing media with or without Lp(a), and with 10% fetal bovine serum, then switched to media with 10% exosome/lipoprotein-deficient fetal bovine serum for 24 h, and media was collected. Conditioned media was then centrifuged at 1,000 times gravity to remove any cell debris and stored at −80°C. Conditioned media samples were processed according to manufacturer's instructions using Nanoview BioSciences (Brighton, MA) tetraspanin capture kits (#EV-RGT-01-V1) that were custom tailored with CD29 capture antibody (BioLegend #303002) to capture both tetraspanin^+^ EVs and CD29^+^ EVs. Samples were diluted (1:2) in the provided kit EV binding buffer. 35 μL of diluted conditioned media sample was incubated overnight on the kit's microarray chips coated with capture antibodies for CD81 (clone JS-81, NanoView Biosciences), CD63 (clone H5C6, Nanoview Biosciences), CD9 (clone H19a, BioLegend), and CD29 (Biolegend, #303002), and negative control IgG (clone MOPC-21, Nanoview Biosciences). Microarray chips were washed three times with EVs binding buffer to remove unbound material. Microarray chips were incubated with fluorescently labeled CD81, CD63, and CD29 antibodies for 1 h. Microarray chips were then washed three times with EV binding buffer, rinsed, dried, and then imaged and quantified using the ExoView R100 platform (NanoView Biosciences). EV capture was quantitively analyzed using ExoView Analyzer 3.0 software (NanoView Biosciences).

### 3D-Collagen Hydrogel EV Calcification

3D-collagen hydrogels were made using rat tail collagen type I (Corning, Corning, NY), at a pH 7–8. 150 μl collagen solution was pipetted on chambered cover glass wells (Thermo Fisher Scientific, LAB-TEK, #1.5 borosilicate) and allowed to solidify in a cell culture incubator for 2 h. Conditioned cell culture media was collected after SMCs and VICs were incubated in M199, BGP, and Lp(a) media with 10% fetal bovine serum for 48 h, which was followed by 24 h in media that contained serum that was depleted in lipoproteins/exosomes. Conditioned media was centrifuged at 1,000 times gravity for 5 min to remove any cell debris. EVs were not further isolated by ultracentrifugation to avoid artifact aggregation prior to adding them to the 3D-collagen hydrogels. 150 μl of media containing one times E^9^ EVs was added to collagen hydrogels and incubated at 37°C, replacing media twice a week for a total of 2 weeks. For calcification assessment, OsteoSense680 (Perkin Elmer; 1:100) was added to the collagen hydrogels on the day prior to image assessment and incubated overnight at 37 degrees Celsius. CNA35-OG488 collagen probe (1:400) was added on the day of imaging and incubated for 1 h at 37 degrees Celsius, followed by washing gels in phosphate buffered saline. Hydrogels were imaged using a confocal microscope A1, and all images were processed with Elements 3.20 software (Nikon Instruments Inc.), and NIH ImageJ software (NIH, Bethesda, MD) was used for OsteoSense680 (calcification) quantification.

### Illustrations

Cartoon illustrations were generated using Microsoft PowerPoint and a licensed Motifolio illustration tool kit (Ellicott City, MD).

### Statistical Analysis

PRISM 9.0 software (GraphPad, San Diego, CA) was used to analyze data by ANOVA with Tukey's multiple comparisons test and Welch's t-test when appropriate. Data were analyzed for normality and equal variance to determine if applied parametric tests were appropriate using Prism software. Bar graph data were plotted in Prism as mean ± standard deviation (STDEV), and individual datapoints were included as dot points on the graphs. For mass spectrometry analysis, *P* values were determined by comparing the relative abundances of proteins identified in the human serum with two or more unique peptides and the results were visualized using volcano plots.

## Results

### Lp(a) Was Taken up by Human Cardiovascular Cells

**Figure 1:**
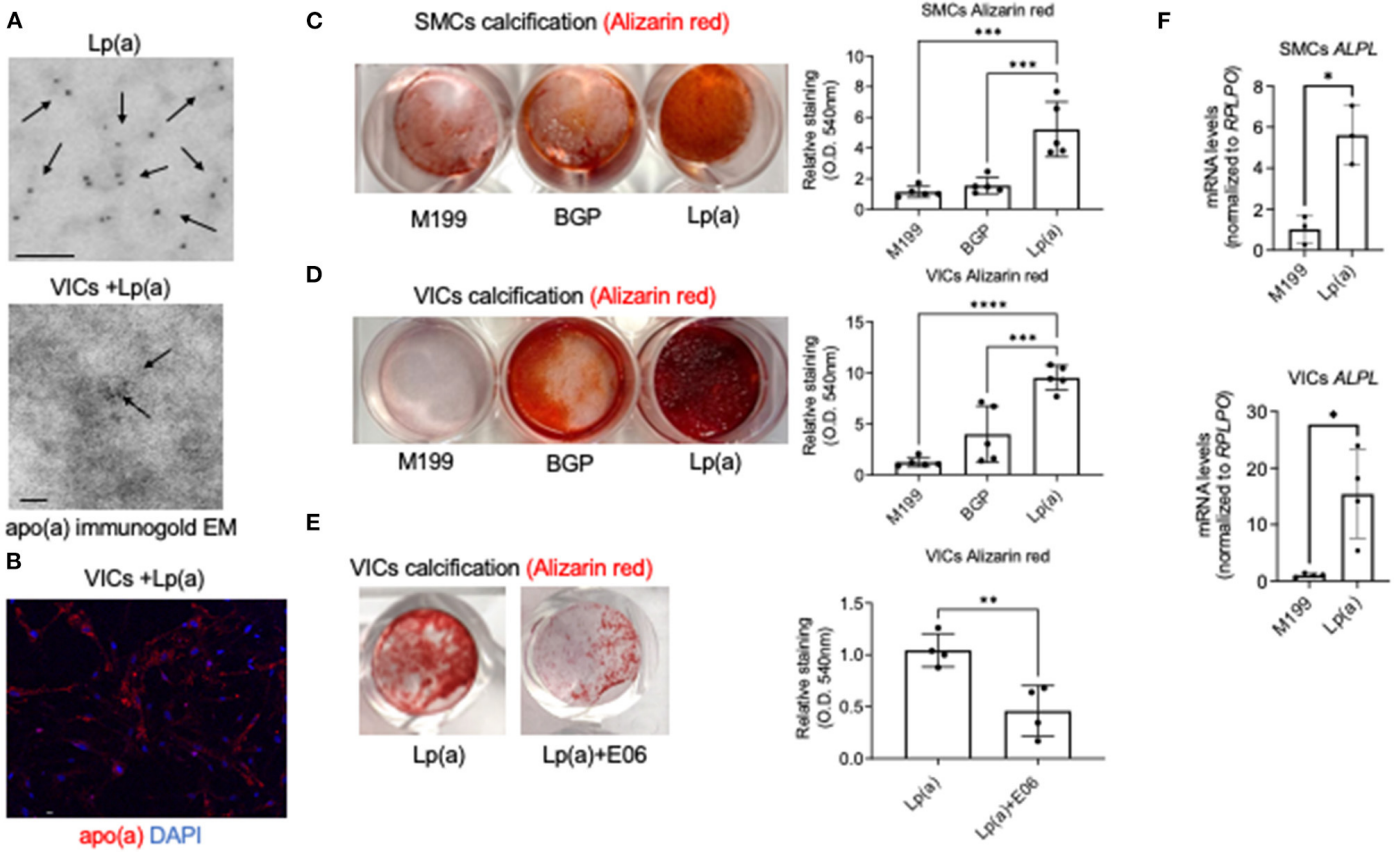
Lp(a) accelerated human cardiovascular cell calcification that was suppressed by E06 antibody. **(A)** Apolipoprotein(a) immunogold electron microscopy of purified human Lp(a) and VICs incubated with Lp(a) (*n* = 6 pooled donors). Arrows indicate examples of positive apolipoprotein(a) immunogold labeling; scale bars = 100 nm. **(B)** Confocal immunofluorescence showing Lp(a) uptake in human VICs (*n* = 5 donors, with an example image shown); scale bar = 20 μm. **(C)** Calcification in human SMCs and **(D)** VICs incubated with control media (M199), or calcifying media containing organic phosphate (BGP) with and without Lp(a), labeled as Lp(a); *n* = 5 donors, analyzed by ANOVA. **(E)** Human VICs incubated as in *B* with the addition of E06 antibody; *n* = 4 donors, analyzed by Welch's t-test. Example Alizarin red (calcification) images shown with quantification included. **(F)** Tissue non-specific alkaline phosphatase (*ALPL*) mRNA levels in SMCs and VICs incubated in control and calcifying media with Lp(a); *n* = 3 donors for SMCs and 4 donors for VICs. Error bars are STDEV, ^*^P < 0.05, ^**^P < 0.01, ^***^P < 0.001, ^****^P < 0.0001.

To assess whether EVs are mechanistically involved in Lp(a) induction of cardiovascular calcification we used a model in which primary human cardiovascular cells were incubated with commercially obtained Lp(a) particles (≥ 95% pure after isolation from human blood). We first performed several validation studies to demonstrate the Lp(a) purity and cellular uptake of Lp(a) with the use of mass spectrometry, immunogold electron microscopy, and confocal immunofluorescence microscopy. To validate the purity of the Lp(a) particles, we performed proteomics and verified that Lp(a) particles purified from human blood contained both apolipoprotein(a) and apoliprotein B, key protein components of Lp(a), as well as multiple other proteins reported to be on Lp(a) (20) (Supplementary Figure 1A; Supplementary Excel File 1). We also validated that commercially purified Lp(a) was similar to that found in human serum by performing proteomics on serum samples from CAVD donors with high Lp(a) (average concentration and STDEV, 214.6 ± 73.1 mg/dL) compared to serum containing low Lp(a) (average concentration and STDEV, 26.5 ± 4.4 mg/dL) (Supplementary Figure 1B). Proteins that were found to be significantly enriched in the high Lp(a) serum samples were found on the purified Lp(a) particles (Supplementary Figure 1B; Supplementary Excel File 1). We further validated the purity of the commercially obtained Lp(a) using a separate method, transmission electron microscopy, in which Lp(a) was immunogold labeled with a validated apolipoprotein(a) antibody. Apolipoprotein(a) immunogold labeled the purified particles (Figure 1A), further validating the Lp(a) purity. We next incubated human valvular interstitial cells (VICs) with Lp(a) and observed apolipoprotein(a) immunogold labeling in these cells (Figure 1A), supporting cellular Lp(a) uptake. Lastly, Lp(a) uptake in human VICs was also observed using confocal immunofluorescence that confirmed cellular Lp(a) uptake (Figure 1B). Together, this data validate the purity of the Lp(a) used in our cell culture experiments as well as showing Lp(a) is taken up by cardiovascular cells.

### Lp(a) Induced Primary Human Cardiovascular Cell Calcification

After validating Lp(a) purity and cellular uptake, we next validated that incubating human cardiovascular cells with Lp(a) induced calcification of primary human vascular smooth muscle cells (SMCs) and VICs. Lp(a) incubation increased calcification induction in primary human SMCs (Figure 1C) and VICs (Figure 1D) cultured in calcifying media containing β-glycerophosphate that provides an organic source of phosphate required for the formation of mineral deposits. Lp(a) contains oxidized phospholipids that can promote inflammation-mediated valve calcification (11), and these pro-inflammatory lipids can be neutralized via E06 antibody in atherosclerotic mice (21). To validate an inflammation mediated mechanism of Lp(a) in promoting calcification, we assessed the ability of E06 antibody to inhibit calcification using primary human VICs as a proof-of-concept. As predicted, E06 antibody suppressed Lp(a)-mediated calcification in VICs (Figure 1E), supporting a plausible mechanistic role of inflammation in Lp(a)-mediated cardiovascular cell calcification. We further validated increased calcification using quantitative PCR. We observed increased mRNA levels of a major calcification inducing gene, tissue non-specific alkaline phosphatase, in human SMCs and VICs incubated with calcification media containing Lp(a) compared to control media (Figure 1F).

### EV Markers and Lp(a) Are in Areas of Calcified Human Cardiovascular Tissue

**Figure 2:**
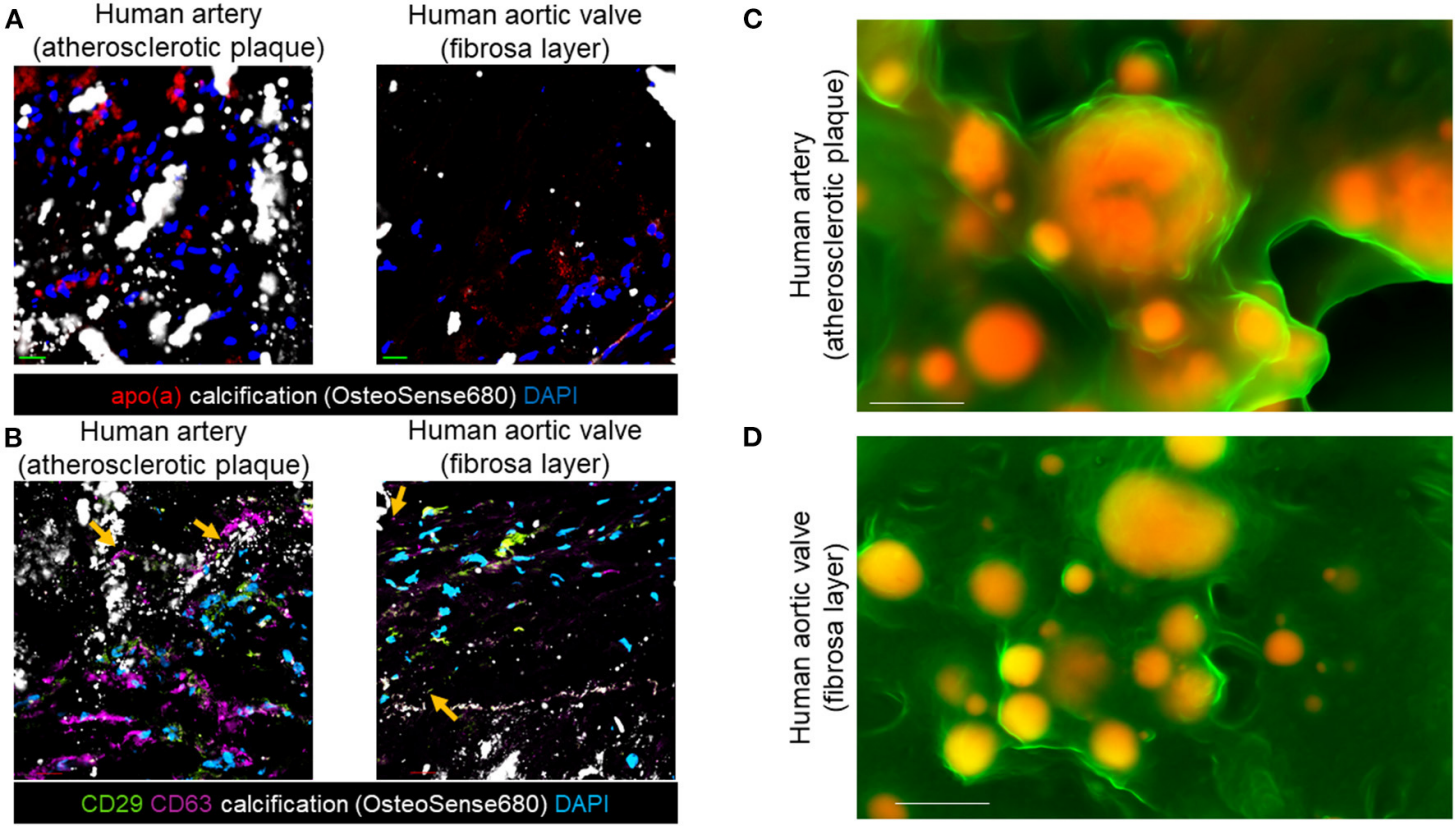
EV markers and Lp(a) localized near calcification in human cardiovascular tissues. **(A)** Apolipoprotein(a) (apo[a]) and OsteoSense680 (calcification) confocal immunofluorescence in human atherosclerotic arteries and human aortic valve tissues (*n* = 5 donors, with example images shown). **(B)** CD29, CD63, and OsteoSense680 confocal immunofluorescence in human atherosclerotic arteries and human aortic valve tissues (*n* = 5 donors, with example images shown). Scale bars = 20 μm. Orange arrows indicate CD29 and CD63 immunofluorescence in acellular regions near microcalcification. **(C)** Density-dependent scanning electron microscopy of calcification (yellow/orange color) in extracellular matrix (green color) of human atherosclerotic arteries and **(D)** aortic valves (*n* = 5 donors, with example images shown). Scale bars = 1 μm.

We demonstrated that inflammation contributes to microcalcification partly via calcifying EVs (8, 9). Therefore, we aimed to assess if pro-inflammatory Lp(a) particles promote microcalcification formation via an EV mechanism. To do this, we first used immunofluorescence and scanning electron microscopy to correlate EVs with calcification in human atherosclerotic arteries and stenotic aortic valve tissues. Protein markers have been established for EVs (22). These markers include CD63, a tetraspanin and marker of an EV subpopulation termed exosomes that are released from multivesicular bodies, and an integrin, CD29 that is found on exosomes as well as on EVs that lack tetraspanins, termed microvesicles, which bud from the plasma membrane. Lp(a) (Figure 2A), CD63, and CD29 (Figure 2B) were observed near calcified areas in human atherosclerotic plaques and aortic valve tissues using confocal immunofluorescence. Notably, CD63 and CD29 were partly observed in acellular areas of calcified human tissues where EVs aggregate (10). We further validated a putative role of EVs in forming cardiovascular microcalcification by using density-dependent scanning electron microscopy to assess calcification morphology in human atherosclerotic arteries and aortic valves. Both human atherosclerotic arteries (Figure 2C) and aortic valves (Figure 2D) contained spherical microcalcification that result from aggregated and calcifying EVs (6, 10).

### Composition of Human Cardiovascular Cell EV Subpopulations Is Altered by Lp(a)

As we observed EV markers (CD63, CD29) of both exosomes and microvesicles in calcified human tissues, we next sought to quantify these two major EV subpopulations in Lp(a)-mediated calcification. For this we used primary human SMCs and VICs, as these cardiovascular cells secrete calcifying EVs (10). We first validated that both cell types released EVs by multiple methods according to the MISEV2018 guidelines (23), including electron microscopy, nanoparticle tracking analysis, and Western blotting. First, we demonstrated the presence of EVs in SMCs and VICs conditioned media by electron microscopy, which showed EVs in conditioned media (Supplementary Figure 2A). Next, we used nanoparticle tracking analysis to show SMCs and VICs released EVs with an average EV size between 150 and 200 nm (Supplementary Figure 2B). We lastly validated the presence of EVs released by SMCs and VICs using Western blotting, which showed conditioned media contained particles positive for EV markers (annexin A1 [ANXA1], CD29, CD63) and absent of cell organelle markers for nucleus, Golgi, endoplasmic reticulum, and mitochondria (Supplementary Figure 2C).

**Figure 3:**
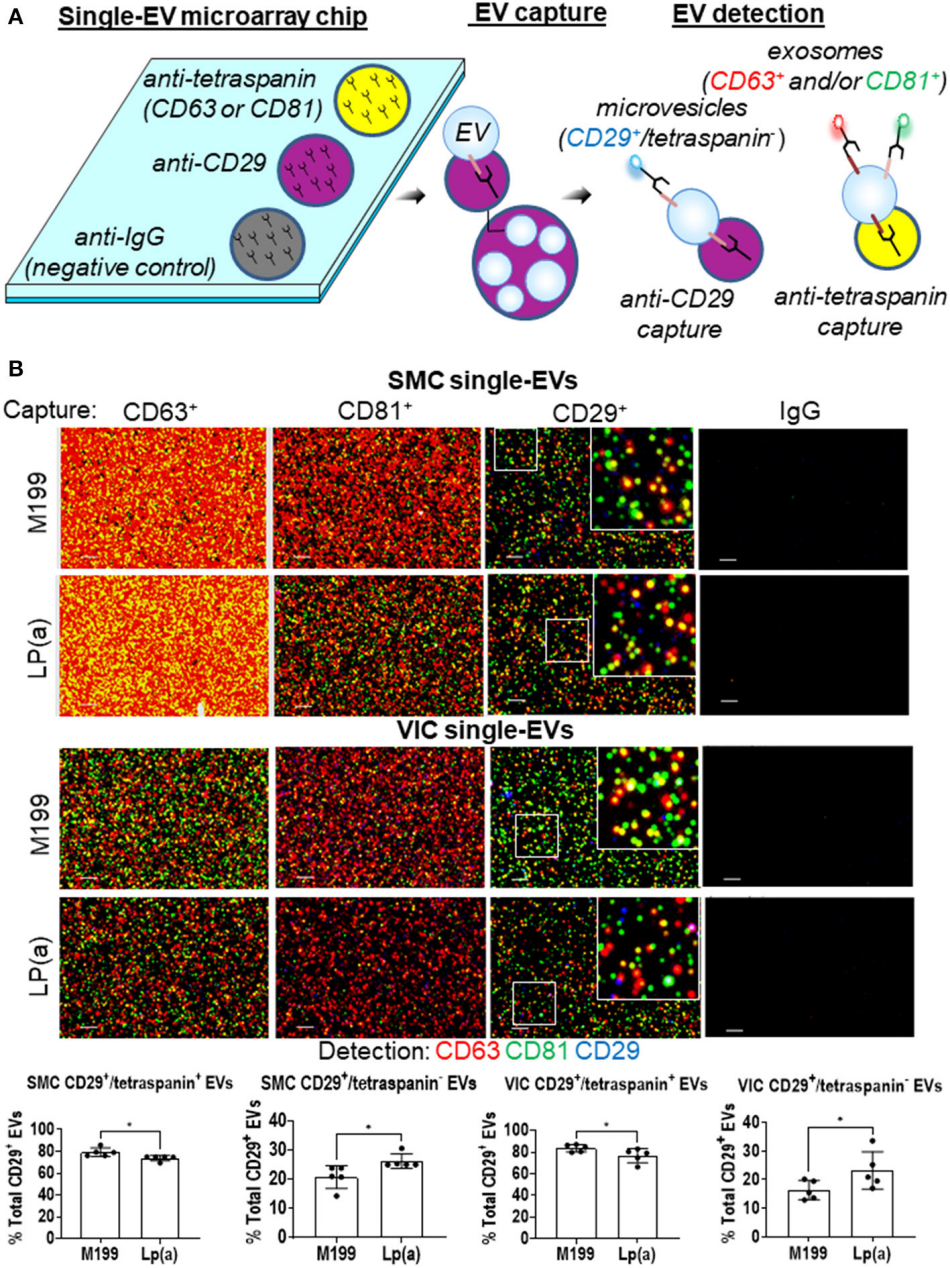
Lp(a) altered EVs composition in calcifying human SMCs and VICs. **(A)** Cartoon depicting the single-EV microarray assay in which microarray chips printed with anti-CD29, anti-tetraspanins, and negative control IgG capture antibodies are used to capture single EVs that are subsequently detected for additional proteins on captured EVs using fluorescently conjugated antibodies. **(B)** Example images of single-EVs captured on microarray chips and fluorescently detected with CD63, CD81, and CD29 or IgG negative control antibodies; scale bars = 1 μm, *n* = 5 donors. White boxes indicates area of the higher magnification inset images. Quantification of CD29^+^/tetraspanin^+^ EVs and CD29^+^/tetraspanin^−^ EVs detected using single-EV microarray and conditioned media from SMCs and VICs incubated in control (M199) or calcifying BGP containing media with Lp(a) (*n* = 5 donors); analyzed by Welch's t-test, *P < 0.05, error bars = STDEV.

After validating human SMCs and VICs release EVs, we next sought to quantify Lp(a) effects on the composition of exosomes and microvesicles, the two major EV subpopulations. We reported that EV composition can be assessed at a single-vesicle level using a single-EV microarray that distinguishes EV subpopulations (10). This method uses antibodies to capture exosomes and microvesicles on microarray chips, which are then incubated with fluorescence antibodies to detect additional proteins on the captured EVs (Figure 3A), after which imaging is used to quantify the vesicle composition. An advantage to this single-EV assay is that it only requires an initial 1,000 times gravity spin to remove cell debris contaminants from EV-containing conditioned media that is then incubated on microarray chips, making its use easier than bulk EV analysis methods that require multiple ultracentrifugation purification steps. Previously, we used ANXA1 as a marker to distinguish microvesicles from exosomes with this method (10). While ANXA1 has been independently demonstrated as a microvesicle marker (22), it is also released from cells as a soluble form and assessment of EVs via capture with annexins is not compatible with calcium chelators found in the microarray EVs binding buffer of commercially obtained tetraspanin capture kits used with this assay. Therefore, we sought an additional marker that would be able to help discriminate microvesicles from exosomes.

Using our previous cardiovascular cell EVs proteomics dataset (10), in combination with an independent microvesicle and exosome proteomics dataset (22), we found CD29 to be a marker of both microvesicles and exosomes. While we were not able to find an additional non-annexin or non-tetraspanin marker from these proteomics datasets that may distinguish these two subtypes by being present largely only on microvesicles, when combined with other protein markers, CD29^+^ EVs can be used to distinguish microvesicles and exosomes. Tetraspanins, including CD63, are found largely on exosomes, and are suggested to be mostly absent from microvesicles (22). While tetraspanins may be on smaller subpopulations of microvesicles (10), they largely serve as an exosomal marker. Therefore, we used tetraspanin markers to distinguish exosomes (tetraspanin^+^ EVs) from microvesicles (CD29^+^/tetraspanin^−^ EVs). We tailored our single-EV microarray assay to capture both CD29^+^ and tetraspanin^+^ EVs. SMCs and VICs were incubated in control or calcifying media with Lp(a), and conditioned media was collected and used with our single-EV microarray assay. We captured exosomes on the microarray chips using tetraspanin antibodies (CD63, CD81) and microvesicles with CD29 antibody (CD29^+^ EVs that do not also contain tetraspanins). We then incubated EVs captured on the microarray chips with fluorescently labeled antibodies for these markers (CD63, CD81, CD29), and used an ExoView R100 platform to image the single-EVs and ExoView Analyzer 3.0 software to quantify the images of captured EVs at a single-vesicle level. This allowed us to assess the EV subpopulations composition under Lp(a) stimulation. Conditioned media from SMCs and VICs incubated with calcifying media containing Lp(a) had about a 5% increase in the number of likely microvesicles (CD29^+^/tetraspanin^−^ EVs), which appeared to come from a shift away from CD29^+^/tetraspanin^+^ likely exosomes (Figure 3B). This data supports that Lp(a) increased release of a likely microvesicle EV subpopulation from calcifying human SMCs and VICs.

### Lp(a)-Mediated Calcification via the Release of Calcifying EVs

**Figure 4:**
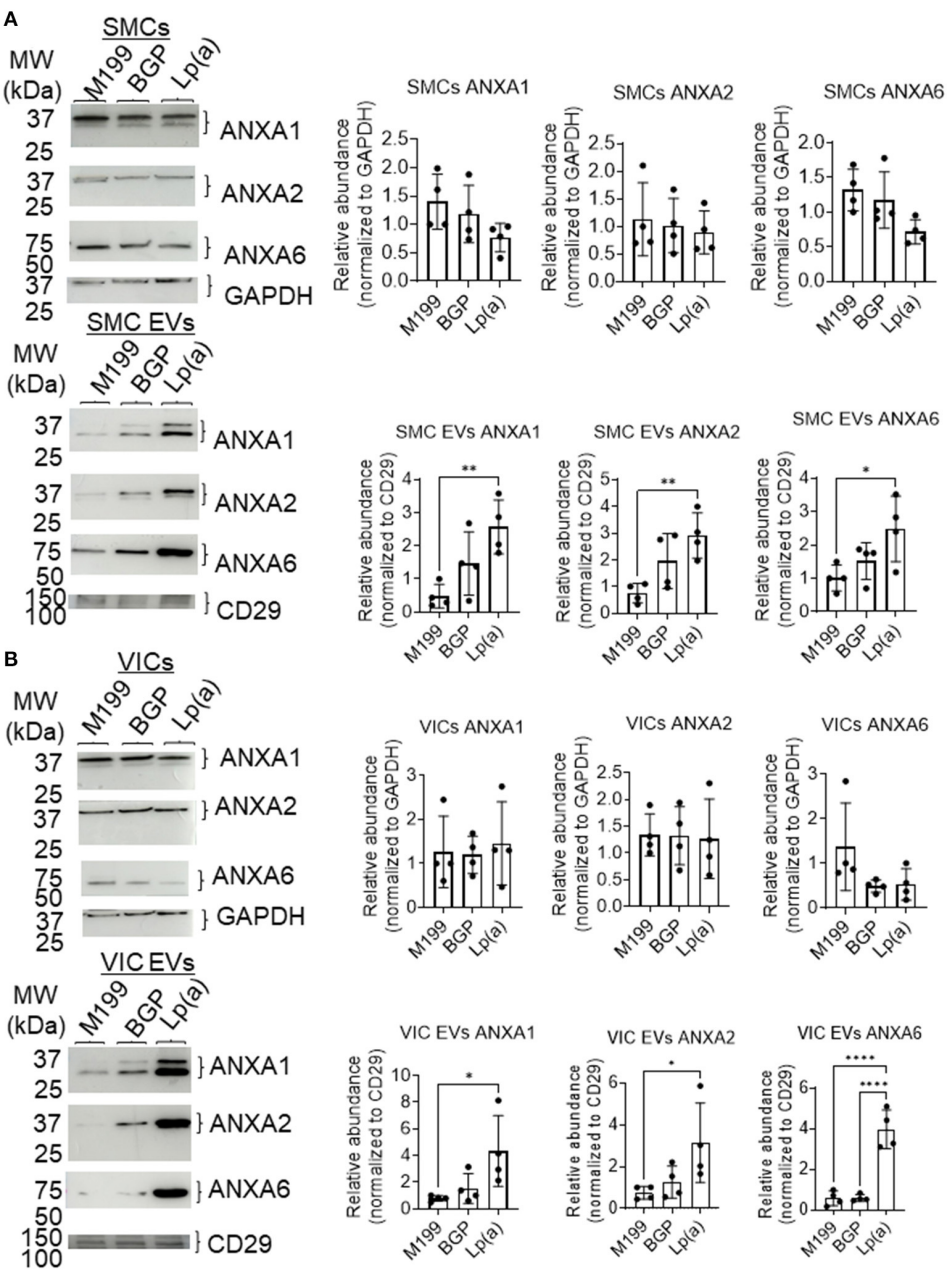
Lp(a) enriched annexins in human SMC and VIC EVs. Western blots with equally loaded (protein abundance) human **(A)** SMCs total cell lysate and SMC EVs lysate and **(B)** VICs total cell lysate and VIC EVs lysate for annexins A1 (ANXA1), A2 (ANXA2), and A6 (ANXA6). Example blots shown, with CD29 and GAPDH used as loading controls for quantification; *n* = 4 donors; analyzed by ANOVA, *P < 0.05, ** < 0.01, **** < 0.0001, error bars = STDEV. Blots were stripped and reprobed for ANXA1, ANXA2 and GAPDH antibodies.

**Figure 5:**
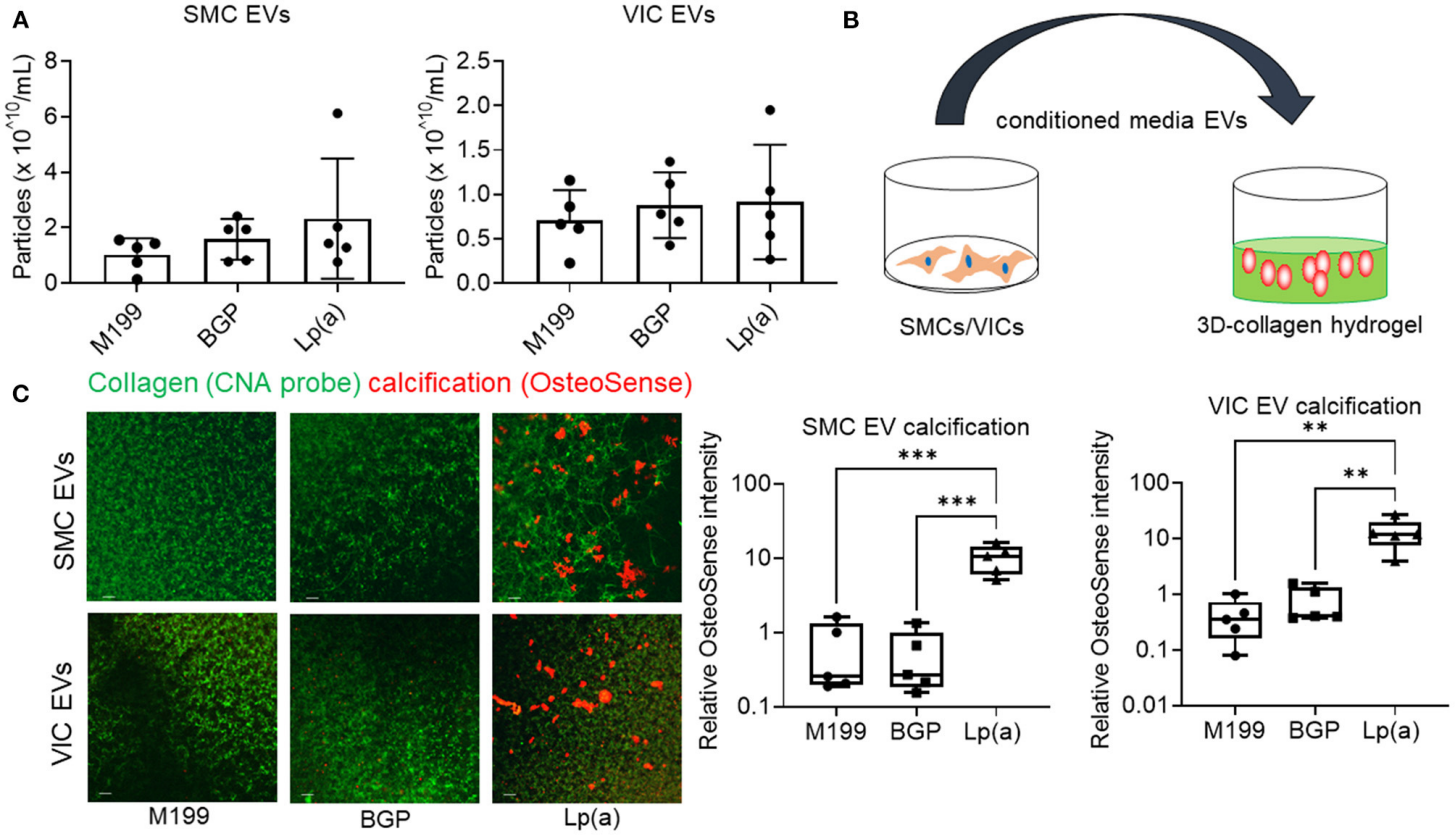
Conditioned media EVs from Lp(a) incubated human cardiovascular cells calcified in 3D-collagen hydrogels. **(A)** Nanoparticle tracking analysis of EVs concentration for human SMCs and VICs cultured in control M199 media, or calcifying organic phosphate containing media (BGP) without or with Lp(a) (Lp[a]); *n* = 5 donors, error bars = STDEV. **(B)** Cartoon of experimental design in which conditioned media containing equal numbers of EVs we transferred to acellular 3D-collagen hydrogels and assessed for calcification potential. **(C)** Confocal immunofluorescence of conditioned media EVs incubated in 3D-collagen hydrogels and stained for calcification with OsteoSense680 (*n* = 5 donors, example images and quantification shown). Analyzed by ANOVA, ** *P* < 0.01, *** < 0.001; graphed as box and whisker plots showing all data points.

Supporting a role of microvesicles in the calcification process, we previously reported that ANXA1^+^ microvesicles can calcify independent of cardiovascular cells in a 3D-collagen matrix (10). We and others have shown that increased annexin abundance on EVs is a marker of calcifying EVs (4, 7, 10). To assess whether EVs released from SMCs and VICs incubated with Lp(a) were calcifying EVs, we first performed Western blot analysis of annexins. Total abundance of annexins A1, A2, and A6 were not significantly altered under calcifying conditions in SMCs (Figure 4A) and VICs (Figure 4B) whole cell lysates; however, Lp(a) increased the abundance of these annexins on SMC EVs (Figure 4A) and VIC EVs (Figure 4B). Lp(a) did not significantly alter the total number of EVs released by calcifying SMCs and VICs in our experimental conditions (Figure 5A). However, altered EV content, including increased annexin protein could increase calcification formation following SMC and VIC Lp(a) incubation. To assess the calcification potential of EVs from SMCs and VICs incubated with Lp(a), we used an *in vitro* calcification assay, in which conditioned media containing equal number of EVs was incubated in acellular 3D-collagen hydrogels (Figure 5B). We have previously demonstrated that this hydrogel model mimics the formation of calcification *in vivo* (6). In this model, calcifying EVs aggregate in collagen matrix and mineralize leading to the formation of microcalcification that coalesces into macrocalcification, a finding that we have validated by confocal, super-resolution, and density-dependent microscopy scanning microscopy techniques (6, 10). EVs from SMCs and VICs incubated with Lp(a) calcified more readily in these hydrogels compared to those from control cells or cells incubated with organic phosphate alone (Figure 5C). This data supports Lp(a) increased the calcifying EV population without altering the total number of EVs in our experimental conditions driving formation of microcalcification.

**Figure 6:**
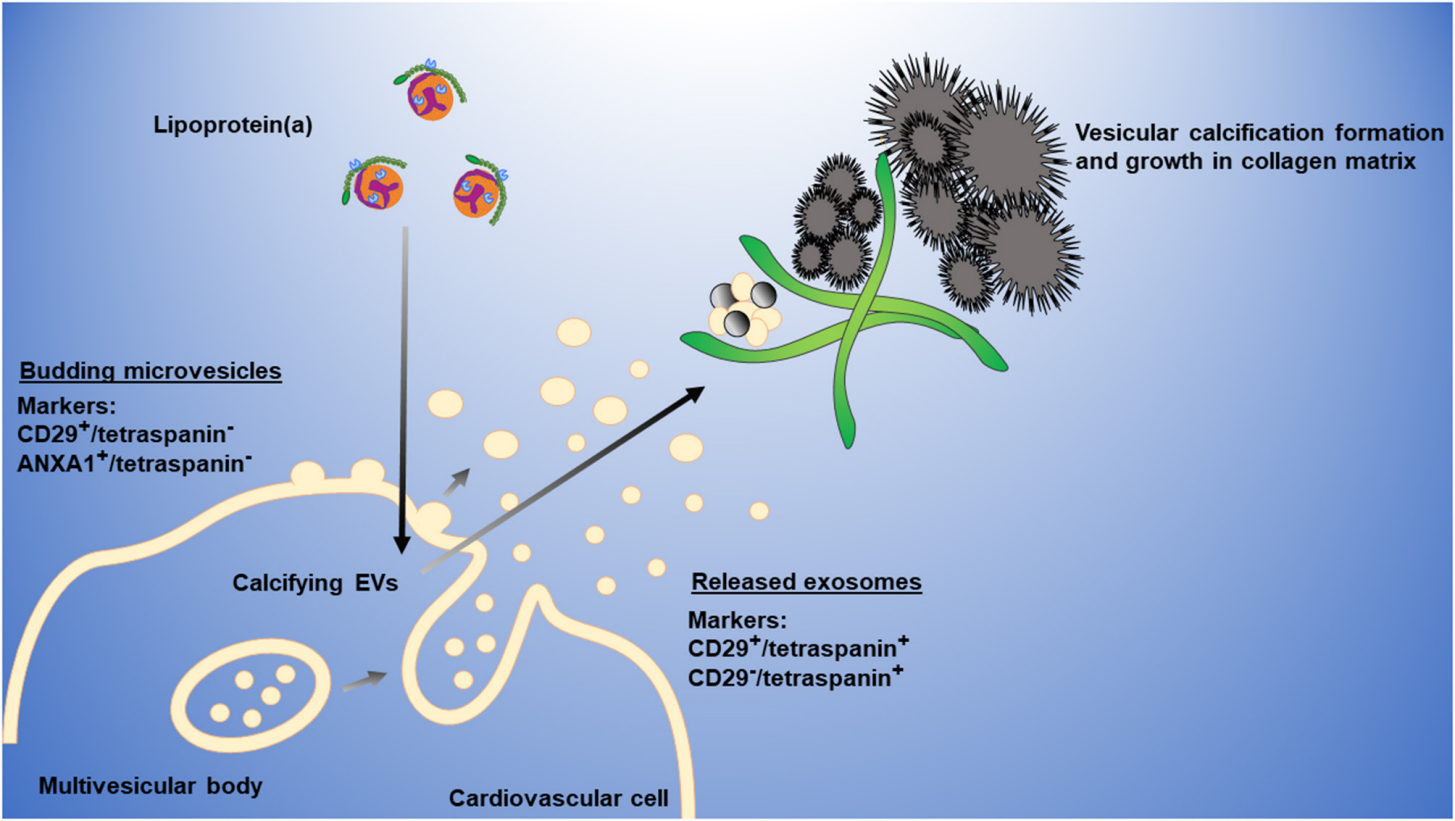
Working model of Lp(a) induction of cardiovascular calcification via EVs. Lp(a) is taken up by cardiovascular cells, stimulating increased release of annexin-enriched calcifying EVs, particularly microvesicles (CD29^+^/tetraspanin^−^ EVs) that get trapped in collagen extracellular matrix, aggregate, and form microcalcification that coalesces into larger macrocalcification. Plasma membrane microvesicles can largely be distinguished from multi-vesicular body-derived exosomes through the presence of markers, including CD29 or ANXA1, and the absence of tetraspanins, including CD63 that is enriched on exosomes.

We propose the following working model to explain how Lp(a) mediates cardiovascular calcification (Figure 6). Lp(a) is taken up by cardiovascular cells stimulating the release of calcifying EVs, which are then trapped in collagen extracellular matrix, aggregate and mineralize. Microvesicles may form a large portion of these aggregates; however, we cannot exclude a role of exosomes in this process, which may also contribute to cardiovascular calcification formation.

## Discussion

Our study has two major novel findings: (1) Lp(a) stimulates release of a cardiovascular EVs subpopulation that calcifies independent of cells in a collagen matrix, which was partially demonstrated *via* (2) a tailored method quantifying EVs at a single-vesicle level. Thus, we identify the release of calcifying EVs as a contributing factor to Lp(a)-mediated pathology, supporting anti-calcific therapeutic potential of targeting Lp(a).

How Lp(a) induces the release of calcifying EVs is unclear, but several related mechanisms have been suggested for Lp(a)-mediated calcification in VICs (24), including inflammatory related pathways involving oxidized lipids and pro-inflammatory proteins like apolipoprotein C3 found on Lp(a) particles (11, 13). Inflammation can alter EVs, and EVs contribute to and reflect inflammatory processes in cardiovascular calcification (8, 9). Lp(a) contains pro-inflammatory oxidized lipids that stimulate cardiovascular calcification (11). Atherosclerotic mice expressing E06 antibody have reduced atherosclerosis pathology as well as reduced valve calcification (21). In agreement, we found that E06 neutralizing antibody suppressed Lp(a)-mediated calcification in primary human VICs, supporting therapeutic potential of neutralizing oxidized lipids in CAVD. In the present study, we propose a mechanistic explanation for how pro-inflammatory particles like Lp(a) may result in increased calcification through inducing release of calcifying EVs that can form mineral deposits in the extracellular matrix of cardiovascular tissues.

Traditionally, EV subtypes are separated using bulk analysis and time-consuming density gradient separation (22). Beyond a single 1,000 times gravity spin to exclude cell debris, our tailored method does not require further sample processing making it more ideal for ease of use. Previously we established a single-EV microarray capable of largely distinguishing microvesicles from exosomes using ANXA1^+^ EVs and tetraspanin^+^ EVs capture (10). ANXA1 was also independently demonstrated as a microvesicle marker (22). However, in addition to being EV associated, ANXA1 is secreted as a soluble protein and is released from EVs with calcium chelation (10), which complicates its use in commercially available vesicle capture assays that utilize calcium chelators. To further tailor our method to capture EVs and distinguish between microvesicles and exosomes at a single-EV level, we incorporated the EV marker CD29, which is found on both exosomes and microvesicles (22). To distinguish exosomes from microvesicles using CD29, we also assessed the presence or absence of tetraspanins on CD29^+^ EVs, with tetraspanin presence indicating that the EVs are likely exosomes and the absence of tetraspanins indicating the EVs are largely microvesicles. While we cannot exclude the possibility of smaller subpopulations of tetraspanin^+^ microvesicles or tetraspanin^−^ exosomes, our tailored method provides an initial step toward better quantitively distinguishing EV subtypes at a single-EV level. Further studies may incorporate additional markers that better define these subpopulations.

Multivesicular body-derived exosomes and plasma membrane-budded microvesicles contain some different cargos that may contribute to disease pathologies, as such understanding how these EV subpopulations each contributes to conditions like cardiovascular calcification is an important area of ongoing research. We found an increase in CD29^+^/tetraspanin^−^ likely microvesicles and a corresponding reduction of CD29^+^/tetraspanin^+^ likely exosomes from cells incubated with Lp(a). While seemingly small by percentage (5%), the total number of vesicles shifting from likely exosomes to likely microvesicles is large considering that in our experimental conditions the number of EVs ranged around 0.5 to 2 times 10^10^/mL. When trapped in collagen these EVs can go on to form aggregates leading to the formation of microcalcifications (10). Why some osteogenic inducing conditions increase the release of calcification-promoting microvesicles is unclear. Microvesicles bud from the plasma membrane, and it is possible this response is due to changes in the plasma membrane composition, or changes in cellular trafficking of proteins to the plasma membrane. Supporting this, we have previously shown that trafficking of sortilin that loads a calcification inducing enzyme, tissue non-specific alkaline phosphatase onto EVs is altered in calcifying SMCs (5). In the present study, we observed increased tissue non-specific alkaline phosphatase in our Lp(a) calcification conditions compared to control media. Additionally, we have shown altered annexin trafficking and increased loading on EVs under calcifying conditions in SMCs and VICs (10), including in the present study.

In conclusion, gaining a better insight of the unique roles of EVs in cardiovascular calcification and how osteogenic differentiation and calcification-inducing stimuli alter release of distinct EVs subpopulations may help identify pathways that could be targeted in the search for anti-calcification cardiovascular drugs.

## Data Availability Statement

The original contributions presented in the study are included in the article/Supplementary Materials, further inquiries can be directed to the corresponding authors.

## Ethics Statement

The studies involving human participants were reviewed and approved by Brigham and Women's Hospital Institutional Review Board. The patients/participants provided their written informed consent to participate in this study.

## Author Contributions

MR, SA, and KZ equally contributed to the concept and collection of data. MR wrote the manuscript. EA contributed to the concept and manuscript writing. EA and MA provided overall supervision and funding for the study. ES provided supervision and contributed to the concept. SC contributed to EVs data collection and analysis. SK and SS contributed to proteomics data collection and analysis. SC and TP contributed to the mRNA analysis. All authors contributed to manuscript editing.

## Funding

This work was supported by research grants from Kowa Company, Ltd. to MA, National Institutes of Health grants (Grant Numbers R01HL136431, R01HL147095, and R01HL141917) to EA.

## Conflict of Interest

The authors declare that the research was conducted in the absence of any commercial or financial relationships that could be construed as a potential conflict of interest.

## Publisher's Note

All claims expressed in this article are solely those of the authors and do not necessarily represent those of their affiliated organizations, or those of the publisher, the editors and the reviewers. Any product that may be evaluated in this article, or claim that may be made by its manufacturer, is not guaranteed or endorsed by the publisher.
