# Supplementary material for: Lipoprotein(a) Induces Vesicular Cardiovascular Calcification Revealed With Single-Extracellular Vesicle Analysis
Source: Front Cardiovasc Med. 2022 Jan 28;9:778919. doi: 10.3389/fcvm.2022.778919 (PMC8831739; doi:10.3389/fcvm.2022.778919)
Supplement: Supplementary file 2 [file Data_Sheet_1.PDF]

Supplementary Material

1 Supplementary Data

Supplementary Excel File 1. Proteomics of purified human Lp(a) and serum from CAVD patients with high Lp(a) (>50 mg/dL) (n = 6) and with low Lp(a) (<50 mg/dL) (n = 3).

1.1 Supplementary Figures

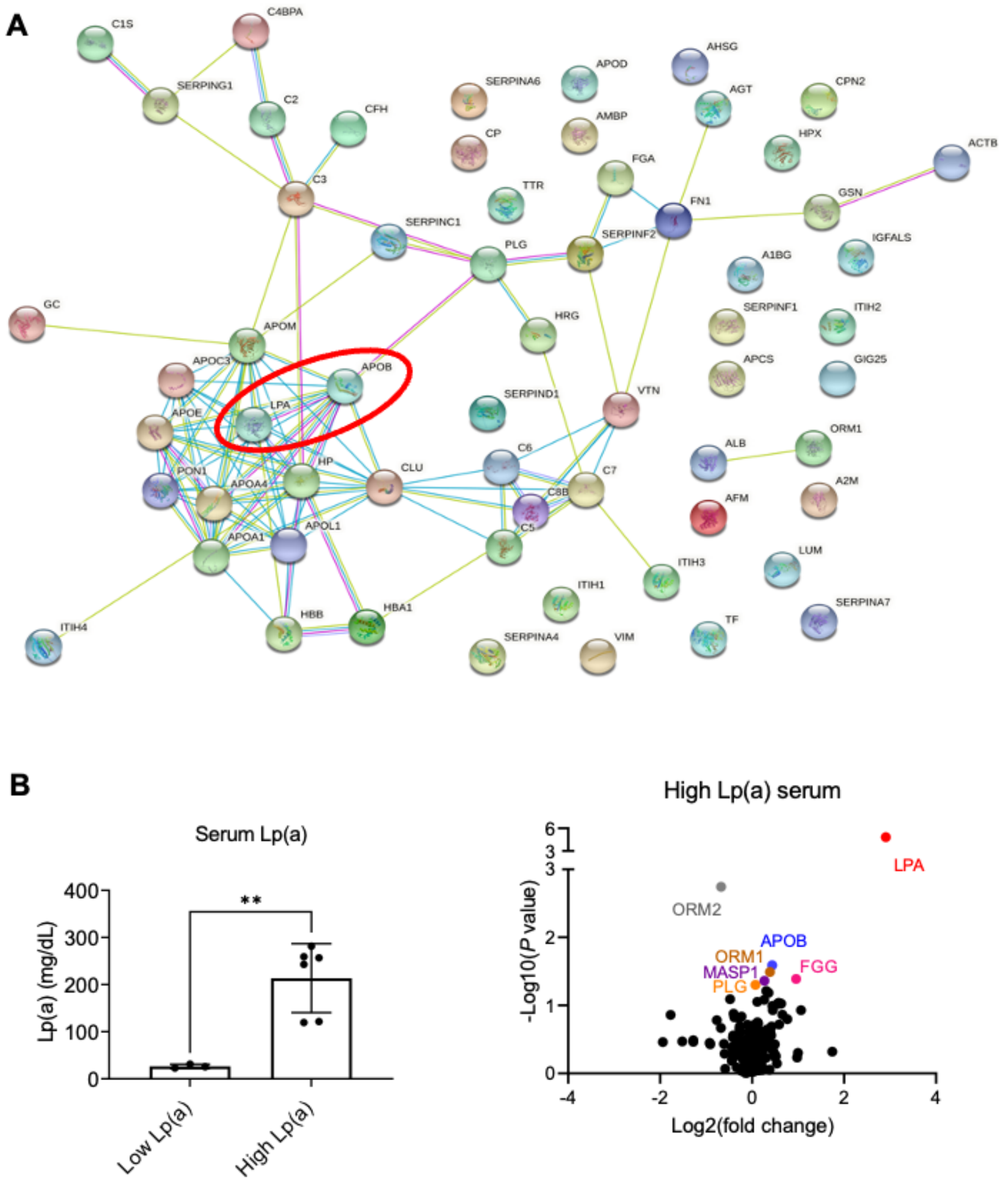

Supplementary Figure 1. Human cardiovascular cells take up Lp(a). (A) String network of proteins identified using unbiased proteomics (3 or more unique peptides) of purified human Lp(a). Red oval indicates core Lp(a) proteins, apolipoprotein B and apolipoprotein(a) (shown with its gene name, LPA). (B) Serum Lp(a) concentrations from donors with high (n = 6) and low (n = 3) serum Lp(a); ***P* < 0.01 analyzed by Welch's t-test. Volcano plot of protein fold change for proteomics (2 or more unique peptides) identified altered proteins in donors with high Lp(a) (n = 6) relative to donors with low serum Lp(a) (n = 3). Significantly altered (*P* < 0.05) proteins labeled. Proteomics datasets included as Supplementary Material Excel File 1.

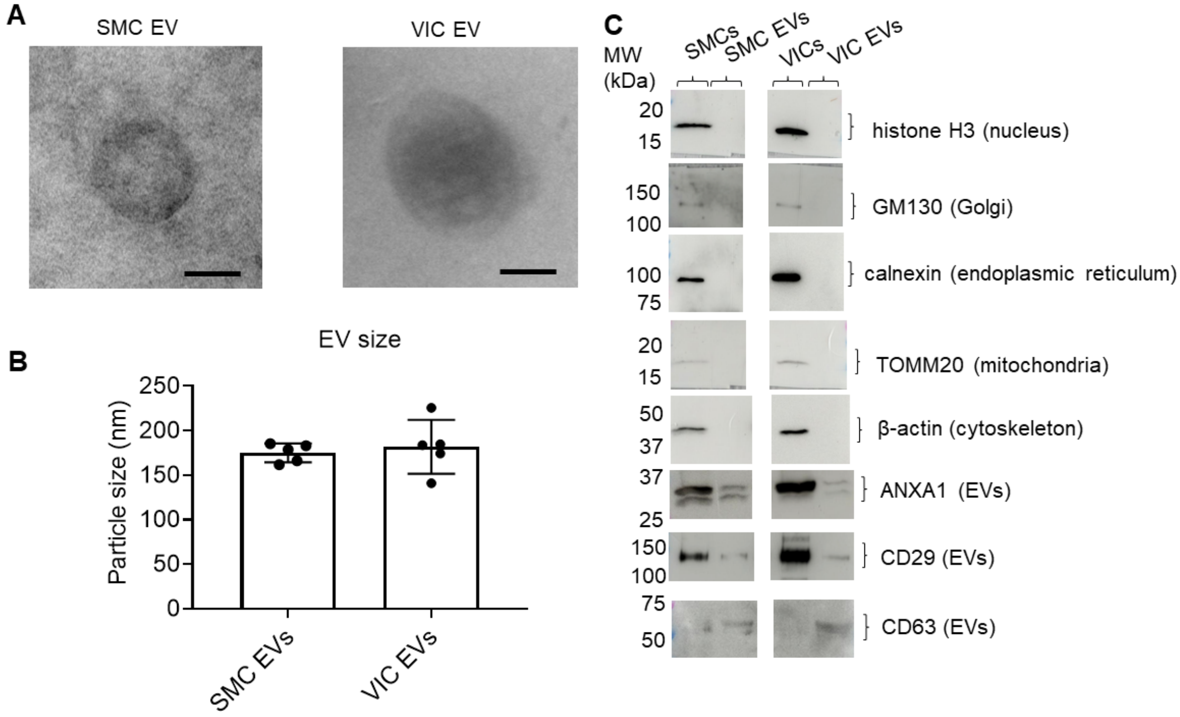

Supplementary Figure 2. Validation of human cardiovascular cell released EVs. (A) Transmission electron microscopy of EVs from human SMCs and VICs conditioned media (n = 3 pooled donors; example images shown, scale bars = 100 nm). (B) Nanoparticle tracking analysis of human SMC and VIC EVs. Mean particle size shown; error bars = STDEV; n = 5 donors. (C) Western blot analysis of cell organelle and EV markers for equal loaded (total protein) SMC and VIC cellular and EV lysates (n = 5 pooled donors).
